# The Impact of Children’s Dietary Habits on the Oral Microbiome: A Systematic Review

**DOI:** 10.3390/nu18162656

**Published:** 2026-08-14

**Authors:** Victor Julien, João Pedro Carvalho, José Carlos Andrade, Célia Fortuna Rodrigues, António Rajão

**Affiliations:** 1 UNIPRO—Oral Pathology and Rehabilitation Research Unit, University Institute of Health Science (IUCS), 4585-116 Gandra, Portugal; a31889@alunos.cespu.pt (V.J.); a29614@alunos.cespu.pt (J.P.C.); 2 UCIBIO—Applied Molecular Biosciences Unit, Translational Toxicology Research Laboratory, University Institute of Health Sciences (1H-TOXRUN, IUCS-CESPU), 4585-116 Gandra, Portugal; jose.andrade@iucs.cespu.pt (J.C.A.); miguelmrajao@ufp.edu.pt (A.R.); 3 Associate Laboratory i4HB—Institute for Health and Bioeconomy, University Institute of Health Sciences—CESPU, 4585-116 Gandra, Portugal; 4 Health Sciences Faculty, Fernando Pessoa University, 4200-150 Porto, Portugal

**Keywords:** microbiota, mouth, child, food, oral health

## Abstract

**Background/Objectives**: The oral microbiome plays a central role in maintaining oral health from early life, with childhood representing a critical period for its establishment and long-term stability. While many environmental factors influence this dynamic microbial ecosystem, diet is distinct in being both universal and highly modifiable. This systematic review aims to evaluate and synthesize current evidence regarding the impact and mechanisms of distinct dietary habits, food matrices, and nutritional components on the composition, diversity, and ecological resilience of the pediatric oral microbiome. **Methods**: A literature review aligned with PRISMA guidelines was conducted via digital searches on PubMed, ScienceDirect, and Cochrane databases (January 2015–December 2025). Search strategies combined MeSH terms and keywords targeting “Microbiota”, “Mouth”, “Child”, “Diet”, and “Oral health”. **Results**: From 1068 records identified, 16 relevant articles met the inclusion criteria. Dietary habits may influence taxonomic and functional profiles. Frequent consumption of sugar-sweetened beverages, sucrose-rich sodas, and sweet treats induces notable dysbiosis and enriches acidogenic/aciduric taxa. Conversely, protective food matrices, including probiotic-fortified dairy products, polyol-based sugar-free chewing gums (xylitol and maltitol), bovine milk, and bioactive-rich agents like green tea, actively suppress cariogenic pathways (specifically *Streptococcus mutans*) and support commensal, health-associated genera without disrupting overall microbial structures. **Conclusions**: Diet represents an important modifiable factor shaping the pediatric oral microbiome, capable of either driving dysbiosis or reinforcing symbiosis. Cultivating a microbiome-informed dietary approach early in childhood supports a resilient microbial architecture, offering a non-invasive, public health framework for long-term oral and systemic disease prevention.

## 1. Introduction

The oral microbiome comprises a complex and dynamic community of microorganisms, including bacteria, fungi, and viruses, that inhabit the oral cavity, representing one of the most diverse microbial ecosystems in the human body with over 700 microbial species, mostly organized into biofilms [1,2]. While “microbiota” strictly defines the microbial community within a specific environment, the “microbiome” encompasses their collective genomes and functional potential [3,4]. This intricate ecosystem is fundamental to maintaining oral homeostasis, initiating digestion, and regulating local immune responses. During childhood, the oral microbiome undergoes critical early development shaped by the mode of delivery, feeding practices, and environmental exposures. Consequently, maintaining a balanced microbial architecture is essential, whereas eubiosis protects the host, microbial dysbiosis acts as a key driver of dental caries, periodontal conditions, and systemic disorders [1,3,5,6].

The implications of pediatric oral dysbiosis extend far beyond the oral cavity, directly influencing global growth, immune maturation, and systemic well-being [7,8]. Recent evidence highlights that oral pathogens can translocate to the gastrointestinal tract, contributing to nutrient malabsorption, which can exacerbate stunting and developmental delays in children [9,10].

Among environmental modulators, diet is one of the major determinants shaping the composition and metabolic function of the pediatric oral microbiome. The oral ecosystem responds dynamically to dietary substrates, particularly fermentable carbohydrates. High and frequent consumption of sucrose has long been established as the principal etiology of dental caries, fueled by its ability to select for acidogenic and aciduric taxa [11]. Sucrose exposure significantly alters the microbial architecture by increasing the abundance of acid-producing species, most notably *Streptococcus mutans* (*S. mutans*), while simultaneously suppressing beneficial taxa, such as *Veillonella* spp., which naturally neutralize organic acids [12,13,14]. Beyond sugars, overall dietary patterns influence oral microbial ecology. Conversely, broader nutritional states also dictate oral ecology; for instance, early-life undernutrition can delay tooth eruption, impair enamel matrix formation, and heighten susceptibility to severe caries and periodontal disease. Thus, dietary choices do not merely influence isolated decay risk but fundamentally modulate the entire oral ecological balance [15,16,17,18].

Dental caries and oral diseases are multifactorial conditions resulting from interactions among the oral microbiome, host susceptibility, dietary habits, oral hygiene practices, fluoride exposure, and other environmental factors. While diet plays a central role in shaping the composition and metabolic activity of the oral microbiome, effective oral hygiene is equally important for controlling dental biofilm accumulation and maintaining microbial homeostasis [19,20,21]. Therefore, dietary influences on the pediatric oral microbiome should be considered within the broader context of these interconnected determinants of oral health.

Investigating the dietary modulation of the pediatric oral microbiome is a vital public health priority [1,22]. Understanding these diet-driven microbial shifts offers a critical opportunity to identify early predictive biomarkers and help design preventive strategies [23,24].

To address existing gaps in the literature, this review aims to evaluate and synthesize current evidence regarding how distinct dietary patterns and components modulate the composition, diversity, and ecological resilience of the oral microbiome in children, providing a framework to guide future non-invasive preventive public health strategies.

## 2. Materials and Methods

### 2.1. Review Guidelines

This systematic review was conducted in accordance with the Preferred Reporting Items for Systematic Review and Meta-analysis (PRISMA) 2020 guidelines [25]. The study protocol was registered in the PROSPERO database (CRD420261406010).

### 2.2. Selection Criteria

Based on the PECO (Population, Exposure, Comparison, Outcome) strategy (Table 1), studies were selected for inclusion if they met the following predefined criteria:

Inclusion criteria:

- Articles published between January 2015 and December 2025;
- Studies involving pediatric populations aged under 18 years old;
- Articles explicitly evaluating the direct impact of dietary habits or nutritional status on the children’s oral microbiome;
- Observational studies (prospective, retrospective, cross-sectional, or cohort designs), randomized controlled trials (RCTs), and qualitative primary studies;

Exclusion criteria:

- Studies evaluating the impact of diet on extra-oral microbial ecosystems (e.g., gut or skin microbiota) without analyzing the oral cavity;
- Systematic reviews, narrative reviews, meta-analyses, conference proceedings, case reports, theses, and dissertations.

### 2.3. Eligibility Criteria

The research question and eligibility criteria were formulated using the PECO (Population, Exposure, Comparison, and Outcomes) framework, which is standard for observational and environmental exposures such as diet. The overarching research question guiding this study was “How do dietary patterns influence the composition and diversity of the oral microbiome in children?” (Table 1).

Because culture-independent, sequencing-based assessments of the pediatric oral microbiome remain scarce, this review also included studies using validated microbiological or biochemical proxies of microbial ecology (targeted bacterial counts, salivary pH, and plaque/gingival indices) where these were the primary outcome reported. Findings derived from compositional/diversity sequencing versus targeted proxy measures are distinguished throughout the synthesis.

### 2.4. Search Strategy

A comprehensive electronic literature search was conducted in the PubMed, ScienceDirect, and Cochrane Library databases to identify relevant articles published between January 2015 and December 2025, yielding a total of 1068 results. The search strategy incorporated Medical Subject Headings (MeSH) terms, including “Mouth”, “Oral Health”, “Microbiota”, “Child”, and “Diet”, combined with relevant free-text keywords (“oral”, “microbiome”, “microflora”, “pediatric”, and “food”). The specific Boolean strings and respective retrieval counts for each database are detailed in Table 2. No language restrictions were applied during the literature search. PubMed, ScienceDirect, and Cochrane Library were selected because, together, they provide broad and complementary coverage of the biomedical, dental, and life-sciences literature relevant to this topic (MEDLINE-indexed journals, Elsevier’s multidisciplinary full-text collection, and the Cochrane trials/systematic-review registers, respectively); this was considered appropriate and balanced to the scope and resources of the present review.

### 2.5. Selection of Articles and Data Collection

The advanced search was conducted using predefined terms. To minimize selection and extraction bias, all records were independently screened by two reviewers (V.J. and J.P.C.), and data extraction was performed in duplicate. Discrepancies between reviewers were resolved through discussion, with a third reviewer (A.R.) making the final decision when consensus could not be reached, ensuring inter-rater reliability throughout the process. The retrieved records from all databases were exported to Mendeley Reference Manager^®^, where duplicate entries were automatically identified and removed. In addition to the electronic database search, backward and forward citation chasing was performed on the reference lists of all studies retained after full-text screening in order to identify additional eligible studies not captured by the database search terms. This citation-based identification step followed the PRISMA recommendations for supplementary search methods and is reported as a distinct pathway in the PRISMA flow diagram.

### 2.6. Quality Assessment and Risk of Bias

The methodological quality and risk of bias of the included studies were independently assessed by two reviewers (V.J. and J.P.C.). For randomized controlled trials, the Cochrane Risk of Bias tool (RoB 2.0) [26] was utilized, with results detailed in Table 3. For non-randomized designs, the Joanna Briggs Institute (JBI) Critical Appraisal Checklists [27] were applied, specifically the tools for cross-sectional studies and quasi-experimental studies. Each item in the JBI checklists was rated as Yes (Y), No (N), or Unclear (U). The detailed JBI appraisal results are presented in Table 4 and Table 5, respectively. Any disagreements between the primary reviewers were resolved through discussion and consensus with a third reviewer (A.R.). A potential limitation is the possibility of publication bias, which occurs when studies with statistically significant or favorable findings are more likely to be published than those with null or negative results. Consequently, the extant literature may exhibit an overrepresentation of positive outcomes, which can lead to an inflation of estimated effects and a reduction in the accuracy of conclusions derived from the evidence base.

## 3. Results

### 3.1. Selection of Articles

**Figure 1:**
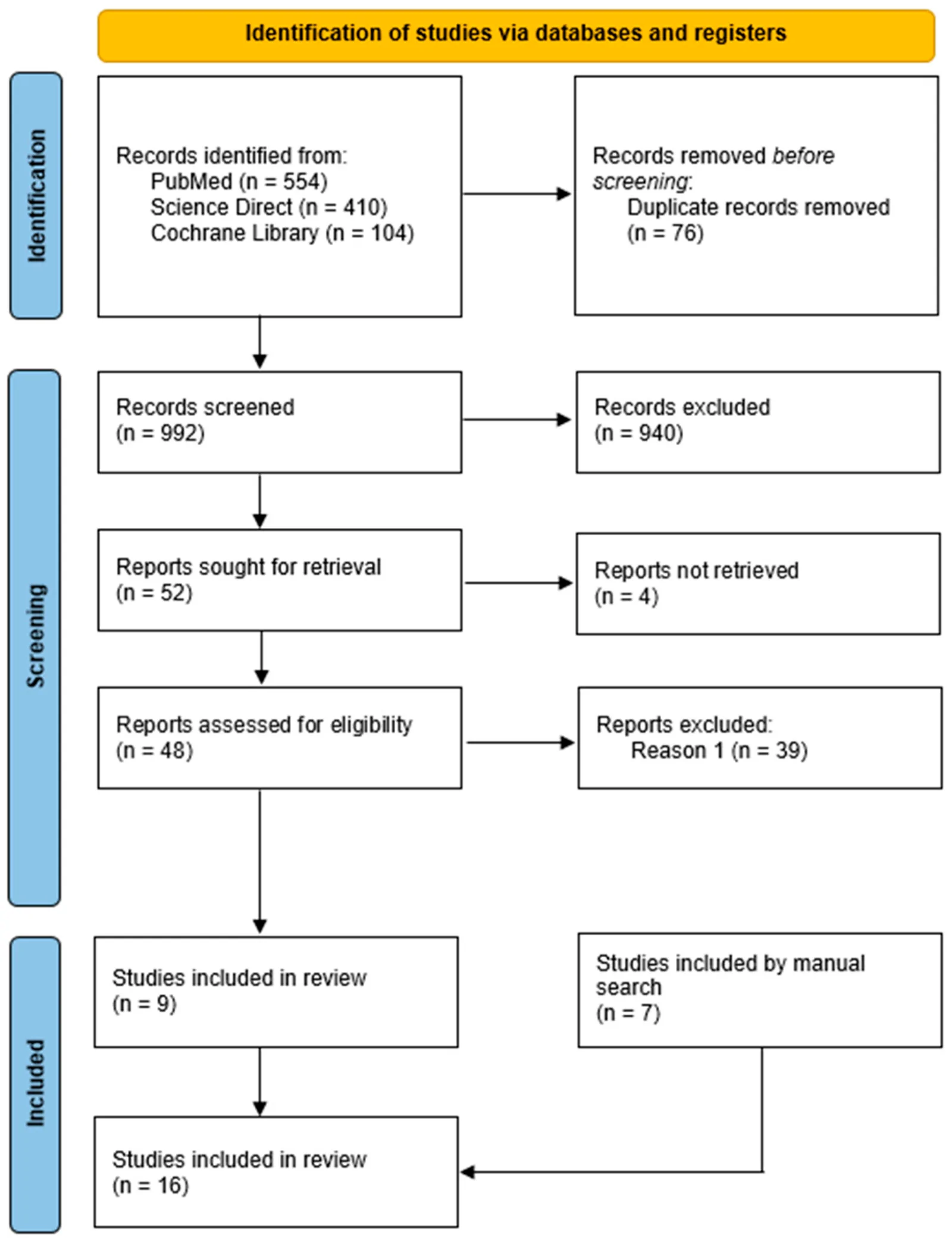
PRISMA flowchart of the studies identified through electronic search. Reason 1: Irrelevant to the topic.

The literature search conducted in the PubMed, Science Direct, and Cochrane Library databases yielded a total of 1068 records, as shown in Figure 1. After removal of duplicates, 992 unique records were screened. After a thorough screening of titles and abstracts, 52 articles were selected for full-text review. Of these, 4 were excluded because they did not provide relevant information for the study objectives. A total of 48 articles were fully assessed, and 9 were included in this review. Additionally, 7 studies were identified through backward and forward citation searching of the reference lists of the included studies, resulting in a final sample of 16 studies. A quantitative meta-analysis was not performed due to clinical and methodological heterogeneity across the included studies, particularly regarding differences in study design, intervention period and dietary exposure.

### 3.2. Sample Characteristics for Study Quality

For the randomized controlled trials, four studies showed a low risk of bias with all criteria fully met: Söderling et al., 2015 [28], Ashwin et al., 2015 [29], Teanpaisan et al., 2023 [30] and Zhang et al., 2023 [31]. In contrast, two studies raised some concerns: Vilela et al. [32], 2020, and Piwat et al. [33], 2020. For the cross-sectional studies, 4 studies presented a low risk of bias, with all evaluation criteria clearly met: Agrawal et al. [34], 2024, Johansson et al. [35], 2018, Chen et al. [36], 2022, and Lommi et al. [13], 2022. Conversely, 3 studies showed a moderate risk of bias due to an unclear exposure measurement and a lack of identification or strategies to deal with confounding factors: Testa et al., 2016 [37], Pang et al. [38], 2022; and Defta et al. [14], 2023. For the quasi-experimental studies, 2 studies showed a low risk of bias with all criteria satisfied: Sarmento et al. [39], 2019, and Dzidic et al. 2018 [40]. Meanwhile, Xu et al. [41], 2021, exhibited a moderate risk of bias. In general, the evidence in this review exhibits a low to moderate risk of bias across the included studies. The quality assessments of the studies are summarized in Table 3, Table 4 and Table 5.

**Table 3:** Risk of bias assessment (RoB 2.0) for randomized controlled trials.

Authors & Year	D1	D2	D3	D4	D5	Overall
Söderling et al., 2015 [28]	+	+	+	+	+	+
Vilela et al., 2020 [32]	!	!	+	!	+	!
Teanpaisan et al., 2023 [30]	+	+	+	+	+	+
Zhang et al., 2023 [31]	+	+	+	+	+	+
Piwat et al., 2020 [33]	+	+	!	+	+	!
Ashwin et al., 2015 [29]	+	+	+	+	+	+

+ Low risk of bias; ! Some concerns; - High risk of bias. D1: Risk of bias arising from the randomization process. D2: Risk of bias due to deviations from intended interventions. D3: Risk of bias due to missing outcome data. D4: Risk of bias in measurement of the outcome. D5: Risk of bias in selection of the reported result.

### 3.3. Characteristics of the Included Studies

For each study included in this systematic review, data were extracted according to specific baseline and clinical parameters. The extracted information encompasses the Authors & Year, Study Design, Objectives, Population, Intervention Period, Dietary Exposure/Intervention, and Main Findings. This comprehensive dataset is systematically organized and summarized in Table 6.

**Table 4:** Joanna Briggs Institute Critical Appraisal Checklist for Cross-Sectional Studies.

	Were the Criteria for Inclusion in the Sample Clearly Defined?	Were the Study Subjects and the Setting Described in Detail?	Was the Exposure Measured in a Valid and Reliable Way?	Were Objective, Standard Criteria Used for Measurement of the Condition?	Were Confounding Factors Identified?	Were Strategies to Deal with Confounding Factors Stated?	Were the Outcomes Measured in a Valid and Reliable Way?	Was Appropriate Statistical Analysis Used?
Agrawal et al., 2024 [34]	Y	Y	Y	Y	Y	Y	Y	Y
Testa et al., 2016 [37]	Y	Y	Y	Y	U	N	Y	Y
Johansson et al., 2018 [35]	Y	Y	Y	Y	Y	Y	Y	Y
Pang et al., 2022 [38]	Y	Y	Y	Y	N	N	Y	Y
Defta et al., 2023 [14]	Y	Y	U	Y	N	N	Y	Y
Chen et al., 2022 [36]	Y	Y	Y	Y	Y	Y	Y	Y
Lommi et al., 2022 [13]	Y	Y	Y	Y	Y	Y	Y	Y

(Y)—Yes, (N)—No, (U)—Unclear.

**Table 5:** Joanna Briggs Institute Critical Appraisal Checklist for Quasi-Experimental Studies.

	Is It Clear in the Study What Is the ‘Cause’ and What is the ‘Effect’	Were the Participants Included in Any Comparisons Similar?	Were the Participants Included in Any Comparisons Receiving Similar Treatment/Care, Other than the Exposure or Intervention of Interest?	Was There a Control Group?	Were There Multiple Measurements of the Outcome Both Pre and Post the Intervention/Exposure?	Was Follow-Up Complete, and If Not, Were Differences Between Groups in Terms of Their Follow-Up Adequately Described and Analyzed?	Were the Outcomes of Participants Included in Any Comparisons Measured in the Same Way?	Were Outcomes Measured in a Reliable Way?	Was Appropriate Statistical Analysis Used?
Sarmento et al., 2019 [39]	Y	Y	Y	Y	Y	Y	Y	Y	Y
Xu et al., 2021 [41]	Y	N	Y	N	Y	Y	Y	Y	Y
Dzidic et al. 2018 [40]	Y	Y	Y	U	Y	Y	Y	Y	Y

(Y)—Yes, (N)—No, (U)—Unclear.

**Table 6:** Data and descriptive analysis of selected scientific articles.

Authors & Year	Study Design	Objectives	Population	Intervention Period	Dietary Exposure/Intervention	Main Findings
**Söderling et al.** **2015** **[28]**	RCT	To determine the effects of xylitol chewing gum on the microbiota of children	73 children	5 weeks	Xylitol chewing gum	- *S. mutans* levels decreased significantly in the xylitol group, in stimulated and unstimulated saliva. - The overall salivary microbiota did not change because xylitol did not alter the global bacterial community structure.
**Vilela et al., 2020** **[32]**	RCT	To evaluate and compare the antimicrobial efficacy of green tea and its main isolated bioactive extract, epigallocatechin-3-gallate (EGCG), as a mouthwash against salivary cariogenic microorganisms in children.	47 children	1 day	Green tea solution, Epigallocatechin-3-gallate extract solution (EGCG), Chlorhexidine (positive control), and Distilled water (negative control)	- Significant reductions (*p* < 0.05) in salivary colony-forming units (CFU) were observed across all treatment groups for both pathogens. - Mutans Streptococci Inhibition: EGCG (79.9%), green tea (68.3%), distilled water (50.6%), and CHX (95.5%). - Lactobacilli Inhibition: EGCG (72.09%), green tea (59.17%), distilled water (41.96%), and CHX (86.02%). - EGCG possesses stronger immediate antimicrobial activity than green tea, but both serve as effective natural alternatives to chlorhexidine (CHX) mouthwashes for children.
**Agrawal et al., 2024** **[34]**	Cross-sectional observational study	To investigate the association of central obesity and habitual food consumption with saliva microbiota and its enzymatic profiles in children.	50 children	-	Sweet treats, dairy products, and plant-based foods	- Clinically relevant differences in central obesity were only modestly reflected in the composition of saliva microbiota (notably the lack of *Pseudomonas guguagenesis* and *Prevotella* spp.). - Habitual consumption of sweet treats was the primary driver of microbial enzymatic reactions, positively correlating with pathways involved in nitrate metabolism induced by *Haemophilus parainfluenzae* and *Veillonella dispar*. - Dairy and plant-based consumption showed significantly lower positive/negative correlation ratios with enzymatic reactions compared to sweet treats.
**Teanpaisan et al.** **2023** **[30]**	Double-blind RCT	To investigate the effect of short-term lozenges containing *Lacticaseibacillus rhamnosus* SD11 on cariogenic pathogens and on oral microbiota in children	121 children	4 weeks of intervention (followed up to 8 weeks)	Lozenges containing *Lacticaseibacillus rhamnosus* SD11	- Probiotic group showed significantly lower levels of *S. mutans* and higher levels of total lactobacilli at 4 and 8 weeks compared to baseline. - 16S rRNA sequencing revealed increased bacterial diversity and beneficial bacteria in the *Firmicutes phylum* and *Bacilli* class, along with a reduction in the mutans streptococci group. - No caries increment or side effects were observed following consumption.
**Zhang et al. 2023** **[31]**	Double-blind placebo-controlled RCT	To investigate the effects of *Lactiplantibacillus plantarum* CCFM8724 on *S. mutans*, *Candida albicans* (*C. albicans*), and oral microbiota of children with early childhood caries (ECC)	Children aged 3–6 years with ECC	28 days of intervention (14 days washout period)	Probiotic *Lactiplantibacillus plantarum* CCFM8724	- Significantly reduced the amounts of salivary *S. mutans* and *C. albicans* (*p* < 0.01). - Increased the abundance of *Firmicutes*, *Granulicatella*, and *Gemella* in dental plaque. - Decreased the abundance of *Proteobacteria*, *Neisseria*, *Bifidobacterium*, and *Catonella*.
**Sarmento et al.** **2019** **[39]**	Longitudinal interventional study	To assess the impact of ‘Petit-suisse’ cheese enriched with probiotics on the dynamics of the children’s microbiota	52 children	28 days	“Petit-suisse” cheese with probiotics (*Lacticaseibacillus casei*)	- Both the control and probiotic products significantly reduced total salivary microorganisms, and although *S. mutans* decreased in both groups, only the probiotic group maintained this reduction after treatment. - The probiotic matrix selectively reduced *Aggregatibacter actinomycetemcomitans* during consumption and kept *Porphyromonas gingivalis* levels significantly low post-treatment.
**Testa et al.** **2016** **[37]**	Cross-sectional observational study	To evaluate the relationship among nutritional status, gingival health, and the composition of oral microbiota in children	45 children (6–14 years old; 20 undernourished, 25 eutrophic)	_	Nutritional status (Undernourished vs. Eutrophic)	- Gingivitis, plaque index, and bleeding on probing were not significantly related to nutritional status (*p* > 0.05). - A significantly higher proportion of undernourished children harbored specific periodontal pathogens and oral bacteria, including *Streptococcus gordonii*, *Capnocitophaga gingivalis*, *Capnocytophaga ochracea*, *Fusobacterium nucleatum* subsp. nucleatum, *P. nigrescens*, *Campylobacter gracilis*, and *T. denticola*.
**Piwat et al.** **2020** **[33]**	Double-blind RCT	To evaluate whether daily probiotic milk prevents caries progression and promotes caries regression in preschool children	487 preschool children (1–5 years old)	6 months	Milk enriched with probiotic bacteria (*Lacticaseibacillus rhamnosus*)	- A significant reduction in levels of *Streptococcus* in the probiotic groups compared to the group of milk without probiotics. - Probiotic milk consumption promotes the arrest and regression of early childhood caries while reducing the salivary load of key cariogenic pathogens.
**Dzidic et al. 2018** **[40]**	Prospective longitudinal observational study	To characterize the development and ecological succession of the oral microbiome in children and assess the influence of postnatal factors and dental caries onset	90 children followed from birth to 7–9 years	Longitudinal follow-up from 3 months to 7 years	Breastfeeding duration, delivery mode (C-section vs. vaginal), and antibiotic exposure	- Ecological succession: *Streptococcus* and *Veillonella* act as early colonizers, while *Neisseria* and *Actinomyces* settle later. - Risk factors: Breastfeeding < 6 months and early antibiotics caused long-term dysbiosis. - Delivery: C-section altered early microbiota but normalized with age. - Caries: Linked to microbial divergence over time and increased *S. cristatus*.
**Xu et al.** **2021** **[41]**	Longitudinal interventional study	To know how daily consumption of probiotic yogurt affects the microbiota in children with caries-free teeth.	30 children (6 completed)	12 months	Probiotic yogurt	- Significant reductions were observed in key potential pathogens, specifically lowering the levels of *Streptococcus* and *Gemella*. - Conversely, a distinct increase was noted in the relative abundance of *Campylobacter*, *Haemophilus*, *Lautropia*, *Bacillus*, *Lactococcus*, and *Solibacillus*. - Probiotic yogurt safely modulates the oral ecosystem by reducing specific cariogenic-associated taxa and enriching diverse commensal genera.
**Johansson et al.,** **2018** **[35]**	Cross-sectional observational study	To explore the relationship between self-reported bovine milk intake and oral microbiome composition and examine links with caries.	154 adolescents	-	Bovine milk	- High bovine milk intake significantly decreased the prevalence and relative abundance of *S. mutans* in both saliva and tooth biofilms. - Despite lower *S. mutans* counts, clinical caries prevalence did not differ significantly between consumption groups. - Milk directly restricts cariogenic pathogen colonization, but its protective effect is neutralized by concurrent sugar intake.
**Ashwin et al.,** **2015** **[29]**	Double-blind RCT	To evaluate the effect of probiotic ice-cream on salivary *S. mutans* levels.	60 children	7 days (6-month follow-up)	Ice cream with probiotic bacteria (*Bifidobacterium animalis* subsp. lactis Bb-12)	- Children consuming probiotic ice cream showed a highly significant reduction in salivary *S. mutans*. - The control group (normal ice cream) showed no significant reduction in bacterial counts. - Probiotic ice cream effectively reduces cariogenic *S. mutans* counts in the short term, but the effect is temporary, indicating that long-term oral health benefits may require regular or continuous dosage.
**Pang et al.,** **2022** **[38]**	Observational comparative study	To identify the characteristics of sugar of the plaque microbiota in adolescents with and without caries.	40 adolescents	-	Sugary food	- Children with low sugar intake and high caries showed a highly cariogenic microbiome, with elevated levels of *S. mutans*, *Lactobacillus*, *Actinomyces*, *S. wiggsiae* (*Streptococcus wiggsiae*), and *C. albicans*. - Children with high sugar intake but caries-free displayed a healthier microbial community, including *C. gingivalis* (*Capnocytophaga gingivalis*).
**Defta et al.** **2023** **[14]**	Cross-sectional observational study	To assess the relationship between diet, dental caries, and the oral microbiome composition in children.	25 children	-	Sugary drinks and snacks	- With frequent sugary drink and snack consumption, Gram-positive bacteria increase, while Gram-negative bacteria decrease. - Sweetened beverages influence oral ecology, increasing the presence of filamentous bacteria and contributing to dysbiosis.
**Chen et al.** **2022** **[36]**	Cross-sectional study	To assess associations between sugar-sweetened beverage (SSB) consumption and oral microbiome diversity in children.	180 children	-	Sweetened beverages	- Higher SSB consumption (≥1 serving/day) was associated with lower oral microbiome richness and diversity. - Children with higher SSB consumption showed decreased abundance of the genera *Fusobacterium*, *Lachnoanaerobaculum*, *Soonwooa*, *Tannerella* and *Moraxella* (*p* < 0.05). - Increased dominance: More SSB intake selectively increases the dominance of aciduric bacteria (*Neisseria* and *Streptococcus*).
**Lommi et al.,** **2022** **[13]**	Cross-sectional comparative study	To compare the composition of the saliva microbiota in children who consume a low or high amount of sweet treats.	453 children	-	Sweet treats	- The high consumption group exhibited a significantly higher abundance of several genera, including *Streptococcus*, *Prevotella*, *Veillonella*, and *Selenomonas* (*p* < 0.05). - Children with high sweet-treat consumption showed higher abundance of several genera associated with oral diseases and lower abundance of genera often associated with a healthy oral environment.

*C. albicans*—*Candida albicans*; *C. gingivalis*—*Capnocytophaga gingivalis*; CFU—colony-forming units; CHX—chlorhexidine; EGCG—epigallocatechin-3-gallate; *S. mutans*—*Streptococcus mutans*; *S. wiggsiae*—*Streptococcus wiggsiae*; SSBs—sugar-sweetened beverages.

## 4. Discussion

### 4.1. Impact of Dietary Sugars on the Oral Microbiome

Evidence from multiple studies suggests that different food categories are associated with either dysbiotic or more favorable oral microbial profiles, providing a framework for understanding how daily dietary patterns may influence pediatric oral health. Although the cariogenic effect of sugar on enamel via acid production is well-established, accumulating evidence shows that carbohydrate intake is associated with alterations in oral microbial homeostasis, driving dysbiosis and amplifying disease risk [14,36,38].

Specifically, high-sugar diets promote the proliferation of acidogenic and aciduric taxa. Defta et al. [14] correlated elevated sugar intake with an increase in Gram-positive, acid-producing bacteria, particularly *Streptococcus* and *Actinomyces*, which act as crucial drivers in early caries development. This is further supported by Chen et al. [36], who reported that children frequently consuming sugar-sweetened beverages (SSBs) exhibited higher abundances of *Streptococcus*, *Gemella*, *Capnocytophaga*, and *Neisseria*, genera favored by substrate availability that participate in acidification and biofilm maturation. Consequently, excessive SSB consumption shifted the pediatric oral microbiome toward lower alpha diversity and altered metabolic activity, rendering the microbial community highly vulnerable to ecological imbalance [36]. Despite the larger sample size of the study conducted by Chen et al. [36], both studies demonstrated analogous outcomes, highlighting the notion that the ingestion of sugar fosters an increase in the presence of acidogenic and aciduric bacteria in school-aged children and adolescents.

However, the included evidence also suggests that the relationship between sugar intake and oral health may depend on the baseline microbial composition. Pang et al. [38] demonstrated that caries risk is associated not only with absolute sugar exposure but also with the ecological configuration and functional potential of the oral microbiome.

These findings suggest that even relatively low sugar intake may contribute to caries development when the oral microbiome is already dysbiotic, although further longitudinal studies are needed to clarify this relationship. Overall, these findings highlight that the relationship between sugar intake and oral health extends beyond enamel demineralization and may also involve interactions with the composition and functional characteristics of the oral microbiome [14,36,38].

### 4.2. Dairy Products and Probiotics in Oral Microbiome Modulation

Probiotic interventions in pediatric populations consistently demonstrate a capacity to modulate the oral microbiome, though clinical efficacy remains highly dependent on the specific strain, dosage, and administration duration [29,33,39,41]. Despite methodological differences among the included studies, their findings consistently support a reduction in cariogenic microorganisms following probiotic supplementation.

Comparing delivery vehicles reveals that targeted lozenge formulations yield comparable, if not superior, ecological modulation relative to probiotic food matrices. While probiotic dairy vehicles like petit-suisse cheese [38] and yogurt [39] effectively reduce *S. mutans* and reshape salivary communities, isolated strain lozenges allow for more targeted local action [30,31]. Specifically, short-term lozenge interventions using *L. rhamnosus* SD11 [30] or *L. plantarum* CCFM8724 [31] not only achieve rapid suppression of *S. mutans* and fungal pathogens like *C. albicans* but also directly expand overall microbial diversity and enrich health-associated *Firmicutes*, *Granulicatella*, and *Gemella*, while suppressing dysbiotic taxa such as *Proteobacteria* and *Neisseria*.

Sarmento et al. [39] reported a sustained reduction in *S. mutans* up to two weeks post-treatment following the consumption of a probiotic ‘petit-suisse’ cheese containing *Lacticaseibacillus casei* by children between the ages of 10 and 12. Following the identification and quantification of the microorganisms present in the saliva samples collected from the volunteers, it was demonstrated that this intervention also selectively reduced periodontal pathogens, including *Aggregatibacter actinomycetemcomitans* and *Porphyromonas gingivalis* [39]. Similarly, Xu et al. [41] observed, in preschool children, that daily consumption of probiotic yogurt containing *Lactobacillus delbrueckii* subsp. *bulgaricus*, *Streptococcus thermophilus*, *Lactobacillus acidophilus*, and *Lacticaseibacillus casei* reshaped the salivary microbiome of preschool children by suppressing cariogenic genera (*Streptococcus* and *Gemella*) while enriching health-associated taxa, such as *Haemophilus*, *Lautropia*, and *Campylobacter* [41].

Comparative studies evaluating milk [33] and ice cream [29] with and without added probiotics demonstrated that significant reductions in *S. mutans* counts occurred exclusively in the probiotic-supplemented groups. These two studies had different dietery exposure times; the milk with *Lacticaseibacillus paracasei* SD1 was given to the volunteers for a time period of 3 months (daily or three days a week), instead of 7 days continuously like demonstrated in the study where the ice cream with *Bifidobacterium animalis* subsp. *lactis* Bb12 and *L. acidophilus* La-5 was used; both studies were randomized and demonstrated that different probiotic strains can have antimicrobial activity against cariogenic bacteria in children aged between 1 and 12 years old [29,33].

Nevertheless, non-inoculated dairy products can contribute to oral homeostasis. Studies have demonstrated that regular milk and dairy consumption may help stabilize the oral microbiome because these foods contain calcium, phosphate, and casein, which buffer acids, protect enamel, and stimulate salivary flow, thereby maintaining a neutral pH [35]. Consequently, probiotic-fortified dairy products, including yogurt, fermented desserts, and kefir, as well as probiotic lozenges, act as effective vehicles for delivering beneficial microorganisms that suppress cariogenic pathogens and restore oral commensal balance [29,30,31,33,39,41].

Furthermore, a systematic review by Carvalho et al. [42] showed that *Limosilactobacillus reuteri* may be able to disrupt pediatric pathogenic biofilms via competitive exclusion and reuterin production. However, its clinical efficacy is strain-specific and transient. Continuous intake and strict oral hygiene are required for sustained benefits [42].

### 4.3. Comparative Impact of Sugar-Free Chewing Gums and Confectionery Treats on the Oral Microbiome

Extensive research highlights a stark contrast between the impacts of sugar-free chewing gums and sugar-sweetened confectionery on the pediatric oral microbiome [28,43]. Söderling et al. [28] demonstrated that xylitol chewing gum significantly reduced *S. mutans* levels in both stimulated and unstimulated saliva, without altering the global bacterial community structure. This indicates that xylitol exerts a targeted antimicrobial effect on cariogenic species while preserving overall salivary microbial homeostasis.

Conversely, studies evaluating sugar-sweetened treats consistently reported associations with a more dysbiotic oral profile. Lommi et al. [13] reported that high sweet-treat intake was associated with significant dysbiotic shifts in the salivary microbiota, characterized by an enrichment of acidogenic and aciduric genera, such as *Streptococcus* and *Prevotella*, which lower oral pH and initiate enamel demineralization. Furthering this evidence, Agrawal et al. [34] found that habitual sweet-treat consumption was associated with microbial enzymatic changes rather than simple taxonomic shifts. This intake positively correlated with pathways involved in nitrate metabolism induced by *Haemophilus parainfluenzae* and *Veillonella dispar*. Crucially, while sweet treats accelerated these enzymatic reactions, dairy and plant-based foods showed significantly lower metabolic correlations, which may be associated with functional shifts that sugar-sweetened treats fundamentally reprogram the functional activity of the oral ecosystem toward disease susceptibility [34].

This divergence emphasizes the therapeutic potential of substituting conventional sugar-sweetened confectionery with polyol-based sugar-free chewing gums as a non-invasive dietary strategy to sustain oral homeostasis in children [13,28,34,43].

### 4.4. Impact of Beverages on the Pediatric Oral Microbiome

Given their liquid formulation, beverages diffuse rapidly across oral surfaces, exerting immediate and widespread effects on the pediatric oral microbial ecosystem. Different categories of beverages, such as soda, carbonated water, artificially sweetened drinks, or green tea, can exert distinct effects on the oral microbiome of children and adolescents [36,44,45].

SSBs and sucrose-containing sodas represent the most detrimental category, triggering an immediate drop in salivary pH that promotes enamel demineralization and stimulates marked bacterial proliferation within dental biofilms [45]. Chen et al. [36] mirrored these findings, demonstrating that children consuming SSBs at least once weekly exhibited reduced oral microbiome diversity. This habit induced significant dysbiotic shifts, characterized by an enrichment of *Streptococcus*, *Gemella*, *Neisseria*, and *Capnocytophaga*, genera heavily implicated in acidification and biofilm maturation, alongside a significant reduction in health-associated taxa such as *Fusobacterium* and *Lachnoanaerobaculum* [36].

Vilela et al. [32] compared a whole green tea mouthwash against its primary isolated polyphenol, epigallocatechin-3-gallate (EGCG), using chlorhexidine (CHX) as a positive control. Both natural formulations induced significant reductions in salivary colony-forming units (CFU); however, EGCG demonstrated superior immediate antimicrobial activity over whole green tea, achieving higher inhibition rates for Mutans Streptococci (79.9% vs. 68.3%) and Lactobacilli (72.1% vs. 59.2%). Although CHX maintained the highest overall bactericidal efficacy, the rapid action of EGCG and green tea underscores their viability as effective, non-toxic natural alternatives to suppress key pathogens and support pediatric oral microbial homeostasis without lowering salivary pH [32].

### 4.5. Impact of Whole Foods on the Pediatric Oral Microbiome

Minimally processed and whole foods can influence the pediatric oral microbiome through a complex interplay between their structural retention, carbohydrate density, and bioactive composition [44,46].

Honey, renowned for its antibacterial, anti-inflammatory, and tissue-healing properties, has been shown to reduce oral mucositis severity and support mucosal repair, indirectly fostering a favorable microbial environment [44,46]. This antimicrobial action is well-documented in monofloral varieties such as Manuka honey (*Leptospermum scoparium*), which leverages a distinct combination of hydrogen peroxide activity and unique phytochemical components to actively compromise the survival of aciduric bacteria [46]. However, despite these therapeutic benefits, honey remains highly rich in simple sugars, necessitating controlled, moderate consumption rather than unrestricted dietary intake.

Although this review focuses on dietary modulation of the pediatric oral microbiome, the effects of diet should be interpreted within the broader context of the multifactorial determinants of oral health. Oral hygiene practices, particularly regular toothbrushing with fluoridated toothpaste, remain fundamental for controlling dental biofilm, maintaining microbial homeostasis, and preventing oral diseases. Therefore, dietary strategies should be considered alongside established preventive oral health measures.

### 4.6. Systemic Host Status and Early Ecological Succession

Beyond direct dietary exposures, host nutritional status and early developmental milestones appear to play an important role in shaping oral microbial resilience. Comparing systemic nutritional states, undernutrition significantly increases host susceptibility to periodontal pathogen colonization (such as *Streptococcus gordonii*, *Fusobacterium nucleatum*, and *Treponema denticola*) compared to eutrophic controls, even when clinical gingival indices remain equivalent [37]. This indicates that host systemic health may influence mucosal vulnerability to subclinical dysbiosis independently of local plaque scores. Furthermore, longitudinal evidence demonstrates that early life events lay the foundation for long-term microbial trajectories [40]. While pioneer colonizers (*Streptococcus* and *Veillonella*) naturally give way to late-settling taxa (*Neisseria* and *Actinomyces*), early environmental disruptions, including breastfeeding under 6 months, C-section delivery, and early antibiotic use, alter this ecological succession [40]. Over time, early divergence in pioneer species like *Streptococcus cristatus* directly correlates with subsequent caries onset, suggesting that early developmental factors and host nutrition modulate how the oral microbiome responds to later dietary habits [37,40].

### 4.7. Limitations and Future Perspectives

The current literature is mainly limited by short-term study designs, parental self-report biases, and inconsistent control of key confounders, including oral hygiene and fluoride exposure. Additionally, many studies do not fully account for the multifactorial nature of oral health, making it difficult to isolate the specific contribution of dietary factors to oral health outcomes. Furthermore, most studies relied on targeted bacterial assays rather than functional metagenomics, overlooking active metabolic pathways. Given that the pediatric oral microbiome evolves dynamically with age, future research must prioritize long-term longitudinal cohorts utilizing high-throughput sequencing. This approach is essential to establish a more robust causality, track microbial trajectories across childhood, and fully elucidate how early dietary patterns shape lifelong oral health.

## 5. Conclusions

Dietary habits appear to be an important modifiable factor influencing the pediatric oral microbiome, influencing the balance between microbial homeostasis and dysbiosis. Current evidence indicates that different dietary patterns and food components can modulate oral microbial composition and function, with important implications for oral and systemic health. These findings support the integration of microbiome-informed nutritional strategies into early pediatric healthcare and caregiver education as a promising, non-invasive approach to promoting long-term oral health. However, further well-designed longitudinal and interventional studies are needed to strengthen the current evidence and support the development of evidence-based dietary recommendations for clinical practice.

## Figures and Tables

**Table 1:** PECO Strategy.

P (Population)	Children and adolescents (aged 6 months to 18 years) without systemic health restrictions
E (Exposure)	Exposure to specific dietary patterns and food consumption habits
C (Comparison)	Comparison between different diets or food types and their effects on the oral microbiome
O (Outcome)	Changes in the composition and diversity of the oral microbiome associated with dietary patterns

**Table 2:** Databases, research strategy and articles retrieved.

Databases	Advanced Research	Articles Found
PubMed	((“Mouth” [Mesh] OR “Oral Health” [Mesh] OR oral) AND (“Microbiota” [Mesh] OR microbiome OR microflora) AND (“Child” [Mesh] OR pediatric) AND (“Diet” [Mesh] OR food))	554
Science Direct	(mouth) AND (oral health) AND (microbiota) AND (child) AND (food)	410
Cochrane Library	((Mouth OR Oral Health OR oral) AND (Microbiota OR microbiome OR microflora) AND (Child OR pediatric) AND (Diet OR food))	104

## Data Availability

No new data were created or analyzed in this study. Data sharing is not applicable to this article.

## Abbreviations

The following abbreviations are used in this manuscript:

CFU	Colony-Forming Units
CHX	Chlorhexidine
EGCG	Epigallocatechin-3-Gallate
JBI	Joanna Briggs Institute
PECO	Population, Exposure, Comparison, Outcome
PRISMA	Preferred Reporting Items for Systematic Reviews and Meta-Analyses
RCT	Randomized Controlled Trials
SSBs	Sugar-Sweetened Beverages
